# Enhancing reading accuracy through visual search training using symbols

**DOI:** 10.1038/s41598-023-31037-5

**Published:** 2023-03-15

**Authors:** Audrey Vialatte, Pierre-Emmanuel Aguera, Nathalie Bedoin, Agnès Witko, Eric Chabanat, Laure Pisella

**Affiliations:** 1 Lyon Neuroscience Research Center, INSERM U1028 CNRS UMR 5292, Trajectoires Team, 16 avenue Lépine, 69676 Bron Cedex, France; 2 Université Claude Bernard, Lyon1, Villeurbanne, France; 3 Laboratoire Dynamique du Langage, CNRS UMR 5596 Lyon, Lyon, France; 4 Université Lumière Lyon2, Lyon, France

**Keywords:** Human behaviour, Neuroscience, Cognitive neuroscience, Visual system

## Abstract

Children with reading disorders present with inaccurate and/or delayed printed word identification. Regarding visual-attentional processing, printed words are letter strings, and each letter is a symbol made of separable features. Simultaneous processing of separable features has been evidenced to be specifically impaired in visual search tasks using symbols in poor readers as well as in a patient with superior parietal lobules (SPL) lesion. Additionally, activation in the SPL has been shown to be abnormally low in dyslexic readers displaying a reduced span of letter strings processing. This deficit has been assumed to impair visual-attentional sampling of printed words. An experiment conducted with 21 dyslexic children tested the hypothesis that a training program based on visual symbol search may stimulate the SPL, leading to a potential benefit transferred to reading performance. We designed the VisioCogLetters serious game and introduced it at random for one month (10 min every day) between four monthly reading sessions. No training was provided between the other (control) reading sessions. Reading accuracy increased without any speed-accuracy trade-off specifically in the session after training. Moreover, the percentage of improvement correlated with the individual time spent at home on training. These results show that improved visual search skills on symbols can translate into enhanced reading performance, and pave a new avenue for future rehabilitation tools.

## Introduction

Developmental dyslexia (DD) is a failure to acquire efficient reading despite normal intelligence and adequate education^1^. According to DSM-5^2^, struggles with accurate and/or fluent word identification and decoding abilities—not with comprehension—characterize this disorder. The most common and sometimes the only causal deficit admitted in DD is a phonological deficit^3,4^. However, researches exploring visual-attentional (VA) processing in reading showed that VA deficits may be present in individuals with DD, sometimes in the absence of phonological impairment^5–7^. They are characterized by a difficulty in symbol visual search^8–10^ in processing multiple alphabetic or non-alphabetic symbols during one ocular fixation (VA span^11,12^), and/or in focusing on spatial relations among hierarchical symbols for global VA processing as opposed to local level processing^13,14^.

While phonological deficits involve the superior temporal area, the supramarginal gyrus and the frontal inferior gyrus of the left hemisphere, the superior parietal lobule (SPL) appears to be the key region for simultaneous visual processing of multiple symbols^15–17^. Indeed, the SPL is bilaterally activated in good readers when several symbols have to be simultaneously processed, but not during the processing of a single symbol^18^. In dyslexics, the SPL is under-activated in both hemispheres when several symbols have to processed simultaneously^19–21^. A neuroimaging study compared two dyslexic adults, one with a phonological deficit but a preserved VA span of letters and one with reduced VA span but good phonological skills. The adult with a phonological deficit showed typical activation of the SPL in a multiple symbols task, but decreased activation of the left hemisphere language areas in a phonological task. On the contrary, the adult with reduced VA span showed typical left inferior fronto-temporo-parietal activation during the phonological task, but decreased SPL activation during the multiple symbols task^16^. This set of neuroimaging studies converged toward a SPL dysfunction in people with reduced VA span, that can be at the origin of their DD. Failure to process many letters simultaneously could prevent from perceiving whole words and identifying them through direct lexical activation. This failure could urge the reader to use mainly analytic grapheme-to-phoneme conversions which are prone to decoding errors in opaque orthographic languages with many irregular words such as English or French. Additionally, with this slow sequential reading procedure, incorrect regular word identification could be prevented only by a large increase of reading time (speed-accuracy trade-off). A training designed to improve simultaneous visual processing of symbols can be assumed to favor the correct decoding of words without affecting reading speed.

Casco and Prunetti^10^ have evidenced slower visual search in poor readers than in good readers only if the target was a multi-featured shape requiring the spatial integration of multiple lines (i.e. symbol made by separable features), but not if it was a single tilted line among single vertical lines. Search time increased in poor readers only if the tilted line was combined with other ones to form a complex symbol-target (e.g. K) among a set of identical complex distractors (e.g. F), whether they were alphabetic or non-alphabetic symbols. Poor readers also performed normally in difficult conjunction searches involving plain objects with non-separable features (e.g. color and orientation). More recently, Khan et al.^22^ made a similar observation in a patient with bilateral SPL lesion recovering from clinical simultanagnosia, a deficit of simultaneous visual processing^23^. Visual search was performed significantly slower by the patient than by controls if the stimuli involved symbols made of a combination of lines (e.g. in a feature-absent task requiring to find a circle among lollipops), but not if plain objects were displayed (e.g. a red disk target among red squares, or a red disk target among red squares and green disks). Using a moving window paradigm^24^, Khan et al.^22^ demonstrated that this slowdown in searching for stimuli made of separable features was associated with a reduced VA field only when facing symbols rather than plain objects. Further investigations conducted in a condition closer to reading, in which all symbols (target but also distractors) were dissimilar, have showed performance reflecting a VA span limited to one single symbol in a patient with SPL lesion^25,26^. Reading is such a condition that implies the simultaneous perception of numerous dissimilar letters made of multiple separable lines, which would explain specific difficulty in reading acquisition in some children with DD who could suffer from SPL-based VA dysfunction. Note that the VA theory of DD could likewise contribute to fulfill the requirement to explain how reading ability can be specifically impaired by a non-linguistic deficit^27^.

**Figure 1:**
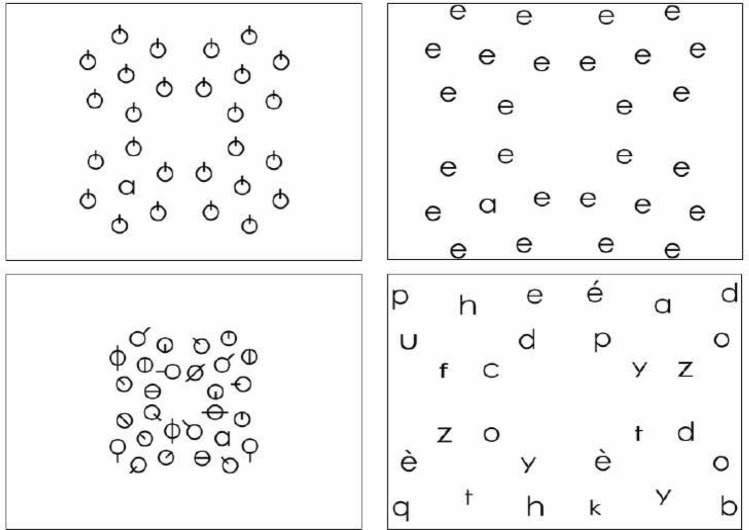
Visual search training using symbols. Four examples of visual displays (presented alone on the entire screen) where the child had to find the target letter “a” among four different types of visual distracters in four blocked training sessions: symbols all similar (top left), letters all similar (top right), symbols all different (bottom left), letters all different (bottom right).

If such specific visual search deficit contributes to DD in some individuals, then a training program specifically targeting visual symbol search should enhance reading performance. Therefore, in the present research, we designed a training program (**VisioCogLetters**, for details see “Methods”, Table 1 and Fig. 1) involving at least 10 min per day of symbol search tasks with increasing difficulty during one whole month, in order to stimulate the VA processing of multiple symbols specifically sub-served by the SPL.

**Table 1:** Visual distracters (symbols or letters, all similar or all different) used for each target letter.

Target	Symbols all different	
a or b or d or p or q or é or è		
c or h or n or u		
f or k or t or v		

Target	Symbols all similar	Letters all similar
a	or	e or o
b	or	d or p
c	or	e or o
d	or	b or q
e	or	a or c
é	or	E
è	or	E
f	or	k or t
g	or	a or q
h	or	n or u
k	or	f or t
n	or	v or u
o	or	c or a
p	or	b or q
q	or	p or d
t	or	f or k
u	or	n or v
v	or	u or n
y	or	z or v
z	or	v or y

**Figure 2:**
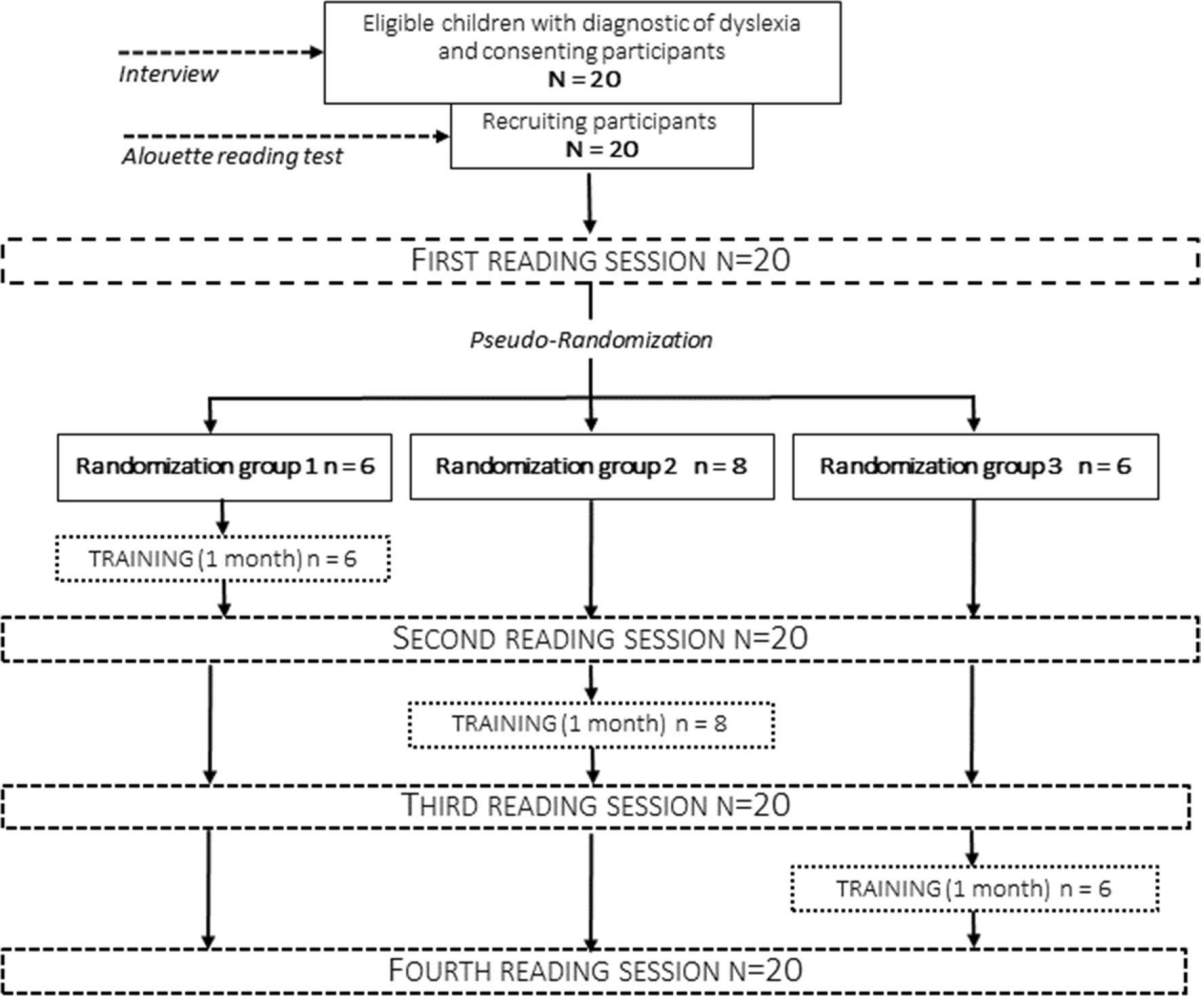
Flow chart illustrating participants’ recruitment and experimental design.

Even if the brain networks^16,17^ involved in phonological and VA deficits are dissociated, they interact during reading acquisition^28^ and most 9–11 years of age children with DD (as in our recruitment) behaviorally exhibit both VA span and phonological deficits^7^. We therefore included into this first study all children with a diagnosis of DD who volunteered to carry out the training program. Twenty 8-to-12-year-old children took part to this study (see on Table 2 their behavioral profile). The experimental design required that their reading ability was assessed through four text reading sessions performed over a three-month testing period (Table 3 and Fig. 2). They were monthly presented with one of four texts equated for reading difficulty regarding each word (**DeltaText**, for details, see “Methods”). The visual search training program was introduced either before the second, the third or the fourth reading test. This experimental design with four reading sessions, within-subject text randomization (see Table 3) and between-subjects training period randomization (Fig. 2) aimed at disentangling the specific effect of the training program from potential effects of text differences (i.e. difficulty) and session repetition (i.e. test–retest) before and after training, to inform about the stability of baseline measures and the maintenance of the training effect, respectively. We expected a significant improvement in reading performance between the text reading sessions immediately preceding and following the month of visual search training, but no difference between control consecutive sessions performed before and after training, nor between texts.

**Table 2:** Behavioral presentation of the sample of children with DD.

Subjects	EVA percentile	EVSP percentile	Chronologic age (month)	Lexical age (month)	Phonological awareness
1	10	25	128	83	0
2	10	27.5	114	92	0
3	20	62.5	109	92	0
4	10	95	124	99	0
5	15	27.5	107	91	1
6	25	17.5	124	99	1
7	15	27.5	138	82	0
8	37.5	62.5	132	104	1
9	5	17.5	127	82	0
10	62.5	27.5	130	99	0
11	37.5	25	136	96	0
12	10	17.5	113	80	1
13	10	27.5	117	92	1
14	10	50	102	82	1
15	5	25	113	87	0
16	5	17.5	111	88	1
17	5	25	120	92	0
18	62.5	17.5	110	87	NA
19	5	75	114	79	1
20	5	17.5	107	90	0

EVA corresponds to the global report task evaluating the visuo-spatial span^44^. EVSP corresponds to a test evaluating visuo-spatial perception of length, size, angle and relative positions^45^. Lexical age was determined with the Alouette reading test^43^. Phonological awareness tasks consisted of suppression, rime or deletion task, 0 means that the child failed at none, 1 that the child failed at least at one of these tasks. NA: data not available from clinical information.

**Table 3:** Randomization of training month (italicized area, with the individual mean and SD of the time, in minutes, daily spent on training) and of the four texts across groups and reading sessions.

Participants	Session 1 Text reading	Month 1	Session 2 Text reading	Month 2	Session 3 Text reading	Month 3	Session 4 Text reading
Group 1		*Training*		*Rest*		*Rest*	
	Pre_T		Post_T				
			Pre_After		Post_After		
					Pre_After		Post_after
S1	Text 1	*4.9 (*± *6.1)*	Text 2		Text 3		Text 4
S4	Text 3	*8.9 (*+ *10)*	Text 2		Text 4		Text 1
S10	Text 4	*6.7 (*± *7.6)*	Text 1		Text 2		Text 3
S13	Text 2	*3.2 (*± *4.9)*	Text 1		Text 4		Text 3
S16	Text 4	*11 (*± *4)*	Text 2		Text 3		Text 1
S18	Text 4	*9.5 (*± *5.4)*	Text 3		Text 2		Text 1

Group 2		*Rest*		*Training*		*Rest*	
	Pre_Before		Post_Before				
			Pre_T		Post_T		
					Pre_After		Post_After
S2	Text 3		Text 2	*12 (*± *14)*	Text 4		Text 1
S5	Text 2		Text 1	*8.1 (*± *5.6)*	Text 3		Text 4
S7	Text 3		Text 4	*9.1 (*± *7.1)*	Text 1		Text 2
S8	Text 1		Text 3	*8.3 (*± *4.9)*	Text 2		Text 4
S11	Text 2		Text 4	*4.5 (*± *5.5)*	Text 3		Text 1
S14	Text 1		Text 2	*3.9 (*± *5.2)*	Text 3		Text 4
S17	Text 2		Text 3	*2.2 (*± *3.9)*	Text 4		Text 1
S19	Text 3		Text 2	*10.4 (*± *7.1)*	Text 4		Text 1

Group 3		*Rest*		*Rest*		*Training*	
	Pre_Before		Post_Before				
			Pre_before		Post_Before		
					Pre_T		Post_T
S3	Text 4		Text 1		Text 2	*8.8 (*± *5.9)*	Text 3
S6	Text 4		Text 3		Text 1	*13 (*± *9.8)*	Text 2
S9	Text 4		Text 2		Text 1	*6.1 (*± *6.3)*	Text 3
S12	Text 2		Text 1		Text 4	*13.3 (*± *10.8)*	Text 3
S15	Text 3		Text 4		Text 2	*8.9 (*± *4.6)*	Text 1
S20	Text 4		Text 2		Text 3	*6.1 (*± *8.3)*	Text 1

## Results

Raw experimental data are provided as open source file (Supplementary Table 1). They consist of individual number of words, number of errors and time needed to read four different texts at the four monthly sessions, which are labelled as pre and post sessions of either the training month or a control month (lying before or after the training month). Reading speed was computed as the total number of words read divided by the time. Reading accuracy was measured as an error rate (see “Methods”).

### Reading speed and accuracy did not differ between texts

Friedman ANOVAs showing no main effect of text neither on reading speed (*F*_*r*_ (N = 20, df = 3) = 0.38, *p* = 0.94; Kendall Coeff. of Concordance = 0.006) nor on error rates (*F*_*r*_ (N = 20, df = 3) = 0.415, *p* = 0.94; Kendall Coeff. of Concordance = 0.007)), as well as matched pairs Wilcoxon tests comparing any pairs of texts (all Zs < 1.15; ps > 0.24), confirmed that the four texts randomly presented for the repeated reading sessions did not differ in difficulty level for our group of dyslexic children. They were thus suitable to reveal potential training effect over test–retest sessions.

### Main effect of sessions’ repetition on reading performance

**Figure 3:**
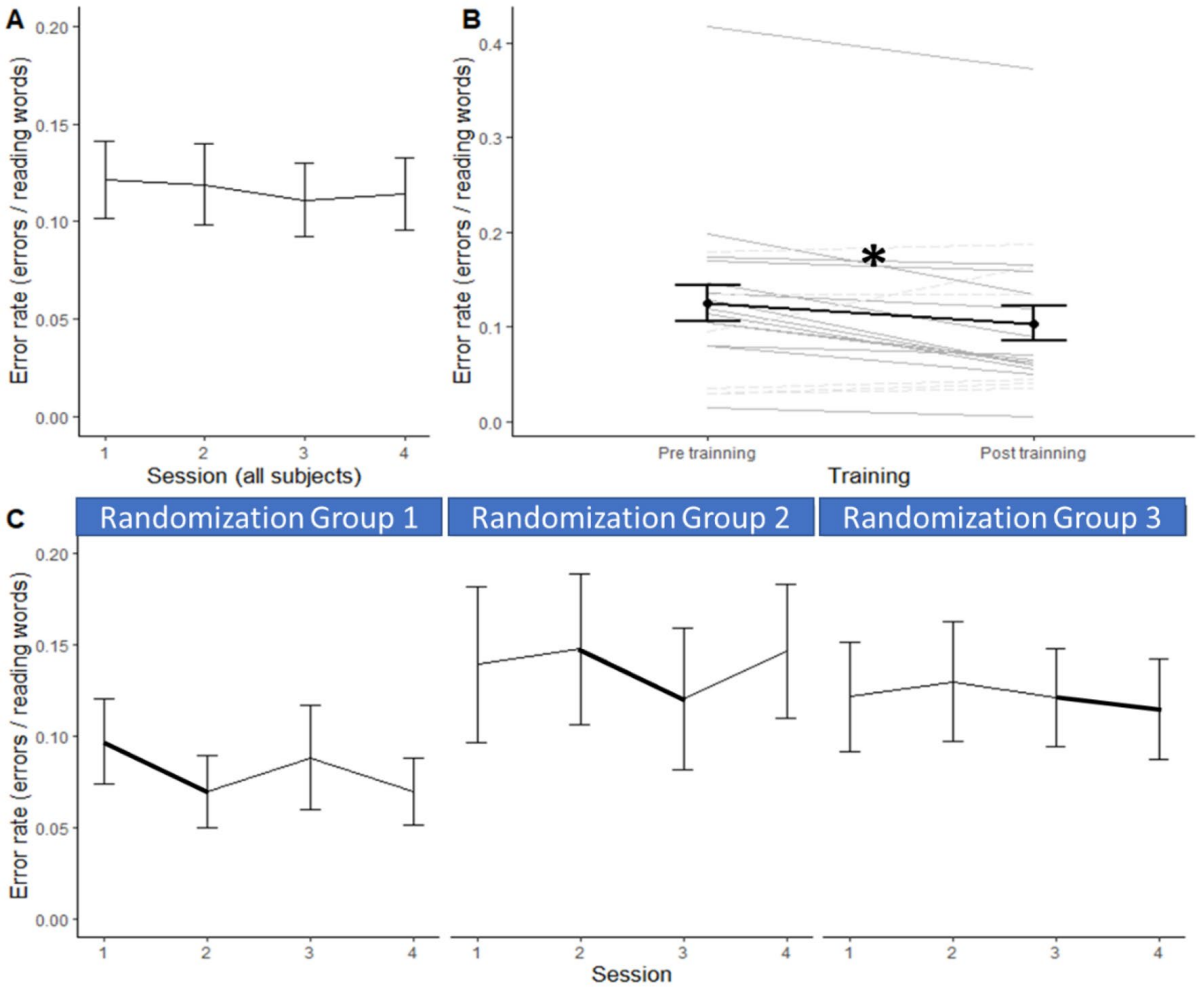
Mean reading error rates and standard errors at the four reading sessions in the whole dyslexic population (**A**) and separately for the 3 randomization groups performing the visual search training at the first, the second or the third month (**C**). Mean error rate just before (pre) and just after (post) the training month in the whole dyslexic group (**B**—in black; and individual data displayed in grey).

**Figure 4:**
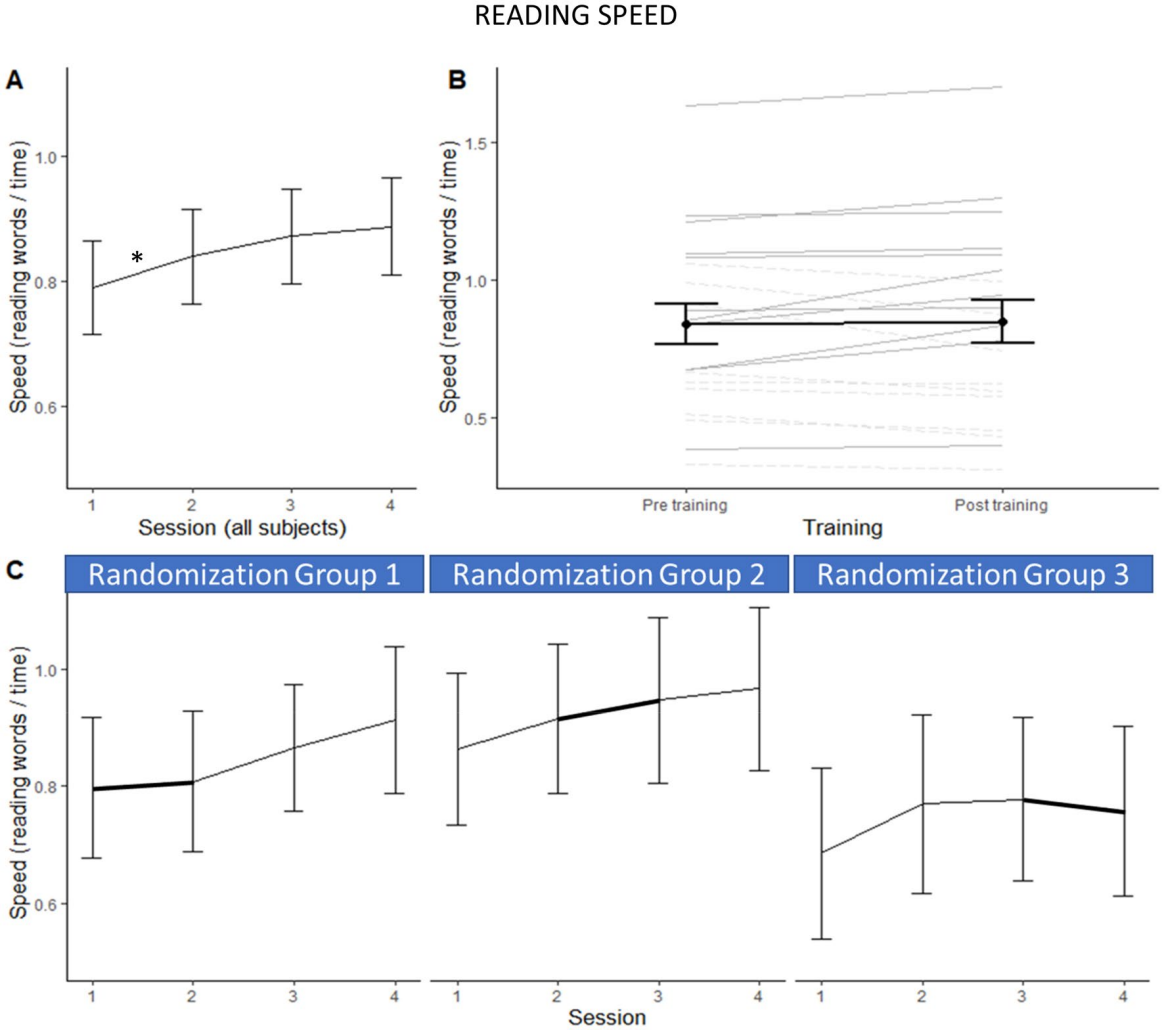
Mean reading speed and standard errors at the four reading sessions in the whole dyslexic group (**A**) and separately for the 3 randomization groups performing the visual search training at the first, the second or the third month (**C**). Mean reading speed just before (pre) and just after (post) the training month in the whole dyslexic group (**B**—in black; and individual data displayed in grey).

When the three randomization groups of children were pooled, Friedman ANOVAs showed no main session effect on reading accuracy (*F*_*r*_ (N = 20, df = 3) = 2,55 *p* = 0.46; Kendall Coeff. of Concordance = 0.043) but reading speed increased over the sessions (*F*_*r*_ (N = 20, df = 3) = 23,08 *p* < 0.001; Kendall Coeff. of Concordance = 0.385). Wilcoxon tests allowed to specify that error rates remained stable between consecutive sessions (all *Z*s < 1.1, *p*s > 0.05; Fig. 3A), reflecting no unspecific test–retest effect on reading accuracy. Reading speed significantly increased between the first and second sessions (*Z* = 2.31, *p* = 0.01), a trend was observed between sessions 2 and 3 (*Z* = 1.57, *p* = 0.06), and no more increase between sessions 3 and 4 (*Z* = 0.56, *p* = 0.57, Fig. 4A). In sum, the children increased their reading speed without decreasing their accuracy over the whole testing period which represents a significant improvement of their general reading efficiency (*F*_*r*_ (N = 20, df = 3) = 24,30 *p* < 0.001; Kendall Coeff. of Concordance = 0.40). This improvement over the three-month period included the specific effect of the visual search training introduced at different times in the three randomization groups (Figs. 3C and 4C).

### Specific effect of VisioCogLetters training

In order to evaluate the effect of symbols visual search training with the same matched pairs Wilcoxon tests as for texts difficulty and for consecutive sessions re-test effects, reading performance measured just before the training session (pre-T) was compared with performance measured just after it (post-T), over the three randomization groups pooled . The unified parameter of reading efficiency displayed a statistical trend towards improvement after training (*Z* = 1.45; *p* = 0.07). This marginal effect was driven by a significant increase in accuracy (*Z* = 2.45,* p* = 0.01, Fig. 3B). Errors rates decreased from pre-training evaluation (*M* = 0.124; *SD* = 0.087, Fig. 4B) to post-training evaluation (*M* = 0.104; SD = 0.082). This reduction of the error rate was of 0.02 error per word, corresponding to an average decrease of 4 errors per text (mean reduction of 16.6%) after the training session. No speed-accuracy trade-off was observed, since reading speed rather increased by 0.01 word/sec with the training, which was not significant (*Z* = 0.41; *p* > 0.05).

**Figure 5:**
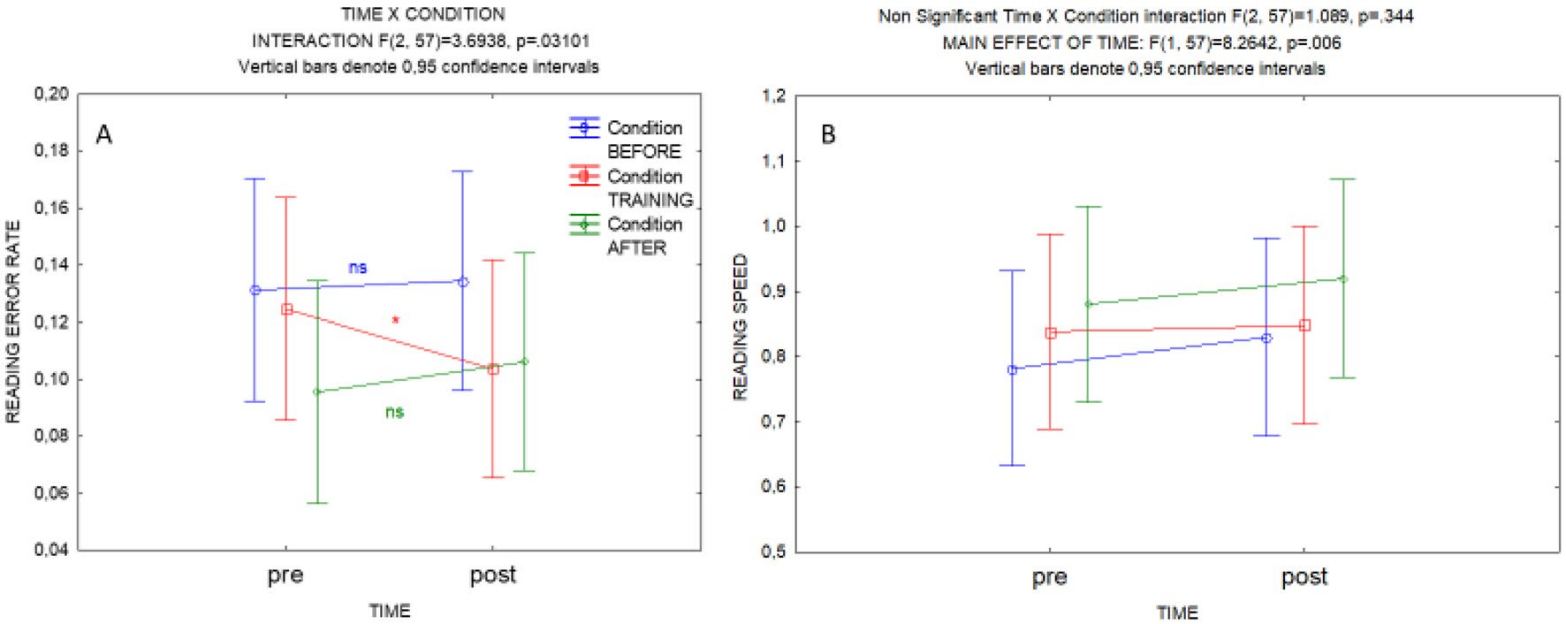
Mean reading error rate (**A**) and speed (**B**) with 0.95 confidence intervals at the reading sessions preceding (pre) and following (post) a month of Training (in red) or a control month lying Before (in blue) or After (in green) training. For error rate (**A**), the significant Time x Condition interaction is mentioned above the graph and the results of the planned comparisons are mentioned on the graph with a star for significant or ‘ns’ for non-significant ones. For speed (**B**), the non-significant Time × Condition interaction is mentioned above as well as the significant main effect of time.

In order to directly test the specificity of the improvement of accuracy during the training month, we also ran a repeated-measures ANOVA with Time (pre, post) as within-subject factor and Condition (Before, Training, After) as between-subject categorical factor. For error rate (Fig. 5A), a significant interaction effect (F(2,57) > 3.6; p < 0.03) was obtained demonstrating that the effect of time between consecutive monthly reading sessions was not the same between conditions over the whole subject sample. Planned comparisons of pre and post reading session error rate within each condition showed a significant decrease only for the Training condition (Months without training BEFORE condition: F(1,57) < 0.15; p = 0.70; Months of TRAINING condition: F(1,57) > 5.96; p = 0.02; Months without training AFTER condition: F(1,57) < 1.51; p = 0.23). For speed (Fig. 5B), no significant interaction effect was obtained (F(2, 57) = 1.09, p = 0.34), only a significant main effect of time (F(1, 57) = 8.26, p = 0.006).

### Compliance effect

**Figure 6:**
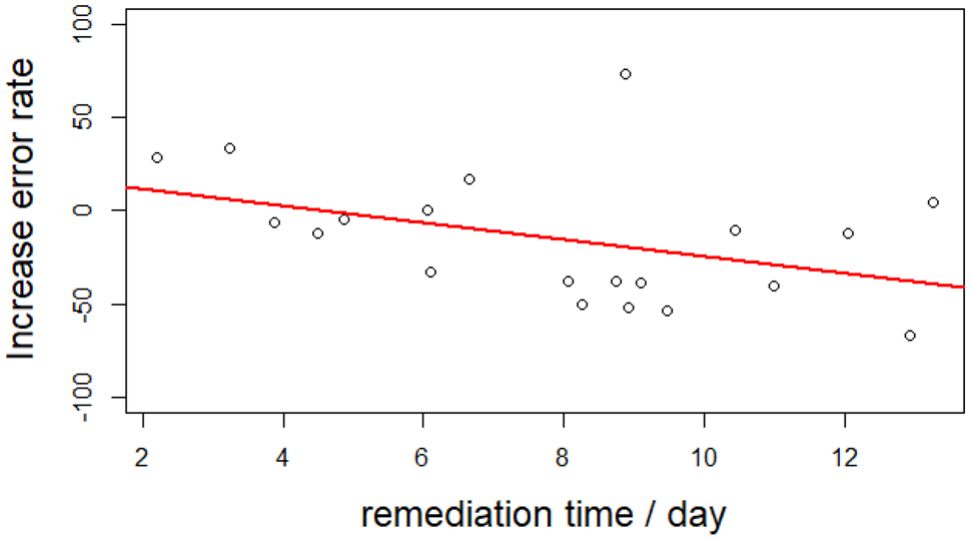
Significant correlation between the time spent per day training visual search on symbols and the decrease in error rate between the pre- and post-training evaluations.

The children did more or less follow the instructions of the 10 min daily home training, both in terms of total time spent on training and in terms of regularity (see on Table 3 the individual mean time spent per day and the standard deviation, respectively), which could have limited the benefit at the group level. The mean time spent performing the visual search training indeed positively correlated with the rate of increase in reading accuracy (Fig. 6; Kendall correlation tau = − 0.37; *p* < 0.05). There was no correlation with reading speed (Kendall correlation tau = − 0.27*p* = 0.098). Note that for several dyslexic children who fulfilled the program with a minimum of 8 min per day on average, the decrease of error rate reached 50%.

## Discussion

The VisioCogLetters training program has been designed based on converging evidence that the VA ability to process simultaneous symbols depends on the SPL whether the task is to search a visual target among distractors^22,25,26^ or to report a series of letters^16,18–20,26^. Its potential effect on text reading performance has been assessed in the present research with a training made at home in full autonomy. On the whole sample, a small but significant gain in accuracy to read aloud from a text was obtained specifically at the reading session following the randomly introduced one-month training and not at those following control months. It corresponded on average to a reduction of 4 errors by text, or a mean percentage of accuracy decrease of 16.6%. Reading speed overall increased across the sessions. Detailed analyses revealed that this overall test–retest effect on reading speed was mainly due to an acceleration between the first and the second sessions, that could reflect the transitory disturbing surprise effect during the first meaningless text reading. Training visual search of symbols was therefore beneficial for reading accuracy with no speed-accuracy trade-off. Importantly, the observed change of reading accuracy correlated to the time that the child spent on training. Six among the twenty participants spent less than 8 min per day on training, while the requested time was a minimum of 10 min per day. This suggests that a better compliance of these children to the training program at home would have increased the size effect.

Future studies specifically dedicated to error decomposition should investigate whether this improvement was achieved by reducing the visual confusions directly trained by the VisioCogLetters exercises, e.g. errors based on mirror invariance or on poor encoding of features and letter locations. Indeed, in the exercises, children were specifically trained at distinguishing the printed letters that remain ambiguous for them from potentially visually confusing distractors made by combinations of similar separable features (Table 1) and at precisely localizing them precisely to point them as fast as possible on the I pad (Fig. 1). More generally, we speculate that visual search training using symbols improved reading performance by stimulating the SPL-based visuo-spatial attention processes underlying printed letter identification and printed word decoding.

It may appear puzzling that reading could be improved by training a visual search task. Many studies have shown that visuo-spatial attention is involved in normal reading^29,30^ and DD^31–33^. Experiments have also shown the transfer of VA training benefits of action video games to reading performance^34–36^. Intervention studies reveal the causal contribution of neurocognitive deficits in DD. The results of our static serious game further support the hypothesis put forward by Antzaka et al.^36^ that SPL-based spatial attention could be a core component mediating this transfer. Indeed, symbol visual search crucially involves the SPL^22,25,26^. Simultaneous visual processing activates the SPL^16,18–20^ and, when measured by the VA span of letters, is linked to reading performance^36,37^. Visual search training using symbols may therefore have strongly requested SPL-based spatial attention, then improved simultaneous visual processing, with positive skill transfer to reading.

Reading is a complex task that involves visual sampling and decoding of sequences of letters that make up each word, which eventually matches with one’s mental lexicon and allows to assign a meaning (i.e., semantics). Since it was unlikely that the visual symbol search training provided in the present experiment could enlarge the mental lexicon of a child, the DeltaText material had been chosen because it contains only regular words, whose identification does not depend on the lexicon as strongly as irregular words. Even for regular words which can be decoded on the basis of extra-lexical rules, French often involves complex grapheme-phoneme decoding rules requiring simultaneous visual processing over at least three letters. Consequently, error rate in DeltaText reading should be dependent not only on phonological abilities, but also on visual-spatial attention, which is specifically impaired after SPL dysfunction, while temporal attention is preserved^38^. We observed no specific gain of our training on reading speed, which might be more dependent on temporal^39^ than on spatial sampling of visual information. We propose that our symbol search training, by stimulating the SPL, allowed to improve simultaneous visual processing of letters. This progress could therefore allow the reader to apply the analytic grapheme-phoneme decoding procedure to larger parts of words, and to favor reliance on the orthographic-lexical direct procedure at least for the small irregular and/or familiar words in DeltaText. Partly due to the instruction to read rapidly, partly due to insufficient mastering of letter identification in our sample, words may have been sometimes guessed by children based on orthographic or phonological lexical neighbor representations. However, this strategy is prone to errors especially when selection in one’s mental lexicon is made based on small sub-units as it is often the case in dyslexic children, and especially in the context of meaningless texts because no semantic anticipation can efficiently participate in guessing. VisioCogLetters training should therefore reduce these risks of error, prompt young readers to rely on both reading procedures, and favor more accurate reading due to improved global visual attention.

A limitation of this study is that the participants have been selected with a diagnosis of DD whatever the kind and proportion of phonological and VA deficits were associated to their reading disability but most of them were lying below the 25th percentile either at the visual attentional span test or at the elementary visuo-spatial perception test (see Table 2). This limits the generalization of the observed benefit to the whole population of dyslexics. Future study should involve a larger sample, and allow us to test separated groups characterized by homogenous behavior at standardized tests designed to evaluate various potential linguistic and cognitive deficits, or by homogenous types of reading errors, in order to determine whether the program is beneficial only for a particular DD profile. It can also be discussed that unlike theoretical research designs using symbol search^22,25^, VisioCogLetters directly trains visual search of letters. Children might then have used verbal labelling^40^ for visual search. Therefore, VisioCogLetters could have also trained verbal recognition of letters and/or have activated letter-phoneme representations used in reading.

Despite these limitations, this study constitutes a first evidence of an improvement of reading accuracy with a letter visual search training and opens several promising perspectives. In terms of rehabilitation, future studies should investigate the DD profile of children that could best benefit from the program. Also, since it was observed that the longer the training per day was, the higher the reading accuracy gain, future studies that would replicate the results should put efforts on the attractiveness of the visual search training that would probably increase the time spent on the serious game and thereby the benefit for reading. Moreover, the training could also be developed for letters bigrams, trigrams or small and frequent words. In terms of prevention for potential reading difficulties, symbol search tasks can easily be performed even as early as 3 years old, before first basic letter and reading acquisition. The parietal cortex seems to play an important role at the beginning of reading acquisition^28^ with an enhanced connectivity with the temporal region which specializes for word recognition in normal reader (visual word form area^41^) but not as well in DD^42^. Enhancing of the simultaneous visual processing activity of this parietal region with the VisioCogLetter game at this crucial period of time should be tested as it might lead more efficient learning acquisition.

## Methods

### Participants

A total of 20 French 8-to-12-year old children (9 females; mean age 10.43 years, SD = 0.94, range = 9 years 1 month to 12 years 1 month) with DD without comorbidities and medication that could affect the central nervous system were involved in this research approved by the ethics committee (CPP Ile de France VI, 2017-A02525-48). This research was conducted in accordance with the relevant guidelines and regulations. Informed written consent was obtained from each participant and their parents. They all displayed normal or corrected-to-normal vision and were free from schedule incompatibility with the involvement in this 3-month follow-up study. The eligibility of children (see Fig. 2) was based on a diagnosis of DD made by a speech therapist. At the time of participant selection, they exhibited a delay of at least 16 months as compared with norms for his/her chronological age in the L’alouette test^43^, which is classically used in France to evaluate the ability to read aloud a meaningless text. We also evaluated whether their visuo-attentional span^44^ and their elementary visuo-spatial perception^45^. They also shared their clinical reports about phonological awareness.

### General procedure

Each child was instructed by the experimenter to read as well as possible one meaningless text, which was different at each monthly home visit over a period of 3 months. The order of the 4 texts was randomly counterbalanced among children. The program of visual search training using symbols was introduced pseudo-randomly during one of the three months research period (3 randomization groups, see Fig. 2) with a I pad (5th generation, model: MP2F2NF/A) lent to the family for one month. During the training month, the child had to daily perform exercises for 10 min at home.

### Visual search training program

Viewing distance to the screen was the one that was comfortable for the child, we required self-adjustment but required that the tablet was on the table and the child was sitting on a chair right in front of the tablet, it was generally about 35–40 cm. The **VisioCogLetters** training consisted in finding a letter among four different types of visual distracters (Fig. 1, Table 1). The target randomly appeared at 10°, 15°, 20° or 25° of eccentricity for 35 cm eye screen distance and the optotype vertical and horizontal size was 1.5° for letter « a » (depending of the letter, for example, the letter « d » was higher due to its vertical bar). The distracters (letters or non-letters symbols) have been selected to be visually as similar as possible to the target letter, i.e. made with the same or similar separable features (see Table 1). For examples, when the target was « b », the similar symbols were alternative combinations of a circle and a line like  or  and the similar letters contained the same features combined in another way (e.g. mirrored letters « d » and « p »). When the target was « n », the similar symbols were alternative combinations of an arch and a line like  or  and the similar letters were « u », « c » or « v ». When the target was « t », the similar symbols were alternative combinations of lines like  or  and the similar letters were « k » or « f ». Other possible visual confusions among letters, like « d » and « a » in which only the length of the line distinguishes the two letters, were trained in the “letter all different” visual displays (Fig. 1, Table 1). Each child was instructed to do 10 min of exercises per day during 4 weeks. Children could start with the letter of their choice at the first level and then choose to change letter or level. Level one was ‘symbols all similar’, level 2 “letters all similar”, level 3 “symbols all different” and level 4 “letters all different” (Fig. 1, Table 1). Next level was available only if the child did finish the previous one. Each level lasted about 3 min so that various letters and types of distracters could be processed each day within a single training session. Each training day, they did not always start with the first level (If the previous day they finished the 3rd level of letter “a” they could start by the 4th level of this letter the next day. The children were required to perform at least 15 letters with the 4 levels completed during the month. If they finished before the end of the month, they had to continue the training 10 min per day until the post-training reading session, it was preconized to do again the 4th level of letters that they found difficult, but they could choose the letters and levels of their choice.

We calculated the mean actual training time spend per day by each child between the pre- and post-training evaluation sessions. These sessions were spaced 29 +/− 3.3 days, and the mean time spend per day on the application performing visual search tasks was 7.9 +/− 3.2 min.

### Repeated text reading measures

The four meaningless 201-word texts used to evaluate the reading aloud performance monthly have been designed by Nathalie Bedoin (**DeltaText**) to be equivalent in terms of number of words, mean orthographic neighbors and mean frequency of neighbors. The word length (letter, phonemes and syllables) and printed word frequency for children (MANULEX^46^) at each specific place was strictly equated through the four texts. The texts were composed of regular words with the exception of short very familiar grammatical words. We measured the time needed to finish to read the whole text, the exact number of words read (because there were possible word or line omissions) and the number of errors. From these measures the reading speed was calculated as the total number of words read divided by the time at which the participant ended the text. Reading accuracy was supplied by error rate, calculated as to the number of errors made divided by the number of words read. A signature of reading performance improvement would increase accuracy without decreasing speed or vice versa. In order to consider one unified parameter of reading performance we computed the efficiency as the number of correct words divided by the reading time in seconds.

### Data analyses

We checked that the four texts read by each child in a randomized order were of equal difficulty in the whole group of participants using multiple dependent samples Friedman non-parametric ANOVA with the text number as the factor, and bilateral matched paired Wilcoxon tests (e.g., to compare texts 1 and 2, we used the reading parameters of the reading session 1 for subjects S1, S8 and S14, of the reading session 2 for subjects S3, S5, S10, S12 and S13, of the reading session 3 for subjects S6, S7 and S9, of the reading session 4 for subjects S2, S4, S11, S15, S16, S17, S18, S19 and S20, and we compared them to the reading session 1 for subjects S5, S11, S12, S13 and S17, the reading session 2 for subjects S1, S2, S4, S9, S13, S14, S16, S19 and S20, the reading session 3 for subjects S3, S8, S10, S15, S18, , the reading session 4 for subjects S6 and S7, see Table 3).

We evaluated unspecific test–retest improvement between consecutive sessions in the whole group of children with DD using multiple dependent samples Friedman non-parametric ANOVA with the session number as the factor, and unilateral matched paired Wilcoxon tests (e.g., to compare sessions 1 and 2, we used the reading parameters of the reading sessions 1 and 2 for all subjects, whatever the text used and whether they were trained with visual symbol search in-between or not).

The specific improvement due to training was statistically evaluated in the whole group of children with DD by unilateral matched paired Wilcoxon tests comparing reading parameters between the consecutive sessions corresponding to the pre- and post-training evaluation sessions for each subject (see Table 3). Pre-training session is the session performed just before the training month (session 1 for the participants of the randomization group 1 for which the training was introduced in the first month, session 2 for the participants of the randomization group 2 for which the training was introduced in the second month, session 3 for the participants of the randomization group 3 for which the training was introduced in the third month). Post-training session was the session performed just after the training month (session 2 for the randomization group 1, session 3 for the randomization group 2, and session 4 for the subjects of the randomization group 3).

Repeated-measures ANOVA was performed with Time (pre, post) as within-subject factor and Condition (Before, Training, After) as between-subject categorical factor for reading error rate and speed (see Supplementary Table 2) to directly and statistically compare training and control conditions.

We also collected the actual time daily spent by each child training visual search at home (see Table 3, italicized area) and tested its correlation with the rate of change in reading performance.

## Supplementary Information

Supplementary Table 1.

Supplementary Table 2.

## Data Availability

The datasets generated and analyzed during the current study are available on supplementary Tables 1 and 2.
