# Supplementary material for: Enhancing reading accuracy through visual search training using symbols
Source: Sci Rep. 2023 Mar 15;13:4291. doi: 10.1038/s41598-023-31037-5 (PMC10017712; doi:10.1038/s41598-023-31037-5)
Supplement: Supplementary file 1 — Supplementary Table 1. [file 41598_2023_31037_MOESM1_ESM.docx]

Supplementary Table 1 : Raw DeltaText data

| Subject | session | text | order | reading words | errors | time | group |
| --- | --- | --- | --- | --- | --- | --- | --- |
| 1 | 1 | 1 | Pre_T | 201 | 35 | 520 | 1 |
| 1 | 2 | 2 | Post_T Pre_after | 181 | 30 | 455 | 1 |
| 1 | 3 | 3 | Post_After Pre_After | 201 | 43 | 425 | 1 |
| 1 | 4 | 4 | Post_After | 201 | 22 | 398 | 1 |
| 4 | 1 | 3 | Pre_T | 201 | 23 | 298 | 1 |
| 4 | 2 | 2 | Post_T Pre_after | 201 | 11 | 258 | 1 |
| 4 | 3 | 4 | Post_After Pre_After | 201 | 7 | 277 | 1 |
| 4 | 4 | 1 | Post_After | 201 | 9 | 257 | 1 |
| 10 | 1 | 4 | Pre_T | 201 | 6 | 163 | 1 |
| 10 | 2 | 1 | Post_T Pre_after | 201 | 7 | 161 | 1 |
| 10 | 3 | 2 | Post_After Pre_After | 191 | 4 | 163 | 1 |
| 10 | 4 | 3 | Post_After | 201 | 3 | 147 | 1 |
| 13 | 1 | 2 | Pre_T | 201 | 6 | 203 | 1 |
| 13 | 2 | 1 | Post_T Pre_after | 201 | 8 | 229 | 1 |
| 13 | 3 | 4 | Post_After Pre_After | 201 | 12 | 189 | 1 |
| 13 | 4 | 3 | Post_After | 201 | 8 | 177 | 1 |
| 16 | 1 | 4 | Pre_T | 201 | 21 | 240 | 1 |
| 16 | 2 | 2 | Post_T Pre_after | 193 | 12 | 204 | 1 |
| 16 | 3 | 3 | Post_After Pre_After | 201 | 19 | 195 | 1 |
| 16 | 4 | 1 | Post_After | 201 | 15 | 215 | 1 |
| 18 | 1 | 4 | Pre_T | 201 | 26 | 303 | 1 |
| 18 | 2 | 3 | Post_T Pre_after | 201 | 12 | 336 | 1 |
| 18 | 3 | 2 | Post_After Pre_After | 189 | 20 | 260 | 1 |
| 18 | 4 | 1 | Post_After | 201 | 27 | 269 | 1 |
| 2 | 1 | 3 | Pre_Before | 201 | 26 | 284 | 2 |
| 2 | 2 | 2 | Post_Before Pre_T | 177 | 24 | 193 | 2 |
| 2 | 3 | 4 | Post_T Pre_After | 201 | 24 | 270 | 2 |
| 2 | 4 | 1 | Post_Before | 201 | 23 | 268 | 2 |
| 5 | 1 | 2 | Pre_Before | 190 | 16 | 234 | 2 |
| 5 | 2 | 1 | Post_Before Pre_T | 201 | 16 | 226 | 2 |
| 5 | 3 | 3 | Post_T Pre_After | 201 | 10 | 224 | 2 |
| 5 | 4 | 4 | Post_Before | 191 | 16 | 204 | 2 |
| 7 | 1 | 3 | Pre_Before | 189 | 31 | 330 | 2 |
| 7 | 2 | 4 | Post_Before Pre_T | 191 | 28 | 304 | 2 |
| 7 | 3 | 1 | Post_T Pre_After | 201 | 18 | 321 | 2 |
| 7 | 4 | 2 | Post_Before | 201 | 26 | 337 | 2 |
| 8 | 1 | 1 | Pre_Before | 201 | 12 | 128 | 2 |
| 8 | 2 | 3 | Post_Before Pre_T | 201 | 24 | 123 | 2 |
| 8 | 3 | 2 | Post_T Pre_After | 201 | 12 | 118 | 2 |
| 8 | 4 | 4 | Post_Before | 201 | 9 | 115 | 2 |
| 11 | 1 | 2 | Pre_Before | 201 | 22 | 242 | 2 |
| 11 | 2 | 4 | Post_Before Pre_T | 201 | 16 | 236 | 2 |
| 11 | 3 | 3 | Post_T Pre_After | 201 | 14 | 194 | 2 |
| 11 | 4 | 1 | Post_Before | 201 | 30 | 215 | 2 |
| 14 | 1 | 1 | Pre_Before | 201 | 24 | 364 | 2 |
| 14 | 2 | 2 | Post_Before Pre_T | 201 | 34 | 298 | 2 |
| 14 | 3 | 3 | Post_T Pre_After | 170 | 27 | 204 | 2 |
| 14 | 4 | 4 | Post_Before | 169 | 33 | 210 | 2 |
| 17 | 1 | 2 | Pre_Before | 201 | 6 | 159 | 2 |
| 17 | 2 | 3 | Post_Before Pre_T | 201 | 7 | 166 | 2 |
| 17 | 3 | 4 | Post_T Pre_After | 201 | 9 | 155 | 2 |
| 17 | 4 | 1 | Post_Before | 201 | 16 | 150 | 2 |
| 19 | 1 | 3 | Pre_Before | 189 | 79 | 318 | 2 |
| 19 | 2 | 2 | Post_Before Pre_T | 201 | 84 | 393 | 2 |
| 19 | 3 | 4 | Post_T Pre_After | 201 | 75 | 465 | 2 |
| 19 | 4 | 1 | Post_Before | 201 | 76 | 324 | 2 |
| 3 | 1 | 4 | Pre_Before | 158 | 11 | 178 | 3 |
| 3 | 2 | 1 | Post_Before Pre_Before | 201 | 13 | 207 | 3 |
| 3 | 3 | 2 | Post_Before Pre_T | 191 | 20 | 176 | 3 |
| 3 | 4 | 3 | Post_T | 201 | 13 | 184 | 3 |
| 6 | 1 | 4 | Pre_Before | 201 | 2 | 183 | 3 |
| 6 | 2 | 3 | Post_Before Pre_Before | 201 | 3 | 163 | 3 |
| 6 | 3 | 1 | Post_Before Pre_T | 201 | 3 | 183 | 3 |
| 6 | 4 | 2 | Post_T | 201 | 1 | 180 | 3 |
| 9 | 1 | 4 | Pre_Before | 201 | 22 | 524 | 3 |
| 9 | 2 | 2 | Post_Before Pre_Before | 201 | 37 | 373 | 3 |
| 9 | 3 | 1 | Post_Before Pre_T | 201 | 40 | 333 | 3 |
| 9 | 4 | 3 | Post_T | 201 | 27 | 349 | 3 |
| 12 | 1 | 2 | Pre_Before | 201 | 33 | 714 | 3 |
| 12 | 2 | 1 | Post_Before Pre_Before | 201 | 44 | 613 | 3 |
| 12 | 3 | 4 | Post_Before Pre_T | 201 | 36 | 606 | 3 |
| 12 | 4 | 3 | Post_T | 192 | 36 | 615 | 3 |
| 15 | 1 | 3 | Pre_Before | 201 | 42 | 447 | 3 |
| 15 | 2 | 4 | Post_Before Pre_Before | 201 | 22 | 432 | 3 |
| 15 | 3 | 2 | Post_Before Pre_T | 201 | 19 | 408 | 3 |
| 15 | 4 | 1 | Post_T | 201 | 33 | 445 | 3 |
| 20 | 1 | 4 | Pre_Before | 191 | 32 | 188 | 3 |
| 20 | 2 | 2 | Post_Before Pre_Before | 170 | 32 | 157 | 3 |
| 20 | 3 | 3 | Post_Before Pre_T | 201 | 27 | 190 | 3 |
| 20 | 4 | 1 | Post_T | 201 | 27 | 202 | 3 |
