# Supplementary material for: Enhancing reading accuracy through visual search training using symbols
Source: Sci Rep. 2023 Mar 15;13:4291. doi: 10.1038/s41598-023-31037-5 (PMC10017712; doi:10.1038/s41598-023-31037-5)
Supplement: Supplementary file 2 — Supplementary Table 2. [file 41598_2023_31037_MOESM2_ESM.docx]

Supplementary Table 2:

|  |  |  |  |  |  |  |
| --- | --- | --- | --- | --- | --- | --- |
| Condition |  | Subj | Error rate PRE | Error rate POST | Speed PRE | Speed POST |
| BEFORE |  | 2 | 0,129353234 | 0,13559322 | 0,707746479 | 0,917098446 |
| TRAINING |  | 2 | 0,13559322 | 0,119402985 | 0,917098446 | 0,744444444 |
| AFTER |  | 2 | 0,119402985 | 0,114427861 | 0,744444444 | 0,75 |
| BEFORE |  | 19 | 0,417989418 | 0,417910448 | 0,594339623 | 0,511450382 |
| TRAINING |  | 19 | 0,417910448 | 0,373134328 | 0,511450382 | 0,432258065 |
| AFTER |  | 19 | 0,373134328 | 0,378109453 | 0,432258065 | 0,62037037 |
| BEFORE |  | 7 | 0,164021164 | 0,146596859 | 0,572727273 | 0,628289474 |
| TRAINING |  | 7 | 0,146596859 | 0,0895522388 | 0,628289474 | 0,626168224 |
| AFTER |  | 7 | 0,0895522388 | 0,129353234 | 0,626168224 | 0,596439169 |
| BEFORE |  | 8 | 0,0597014925 | 0,119402985 | 1,5703125 | 1,63414634 |
| TRAINING |  | 8 | 0,119402985 | 0,0597014925 | 1,63414634 | 1,70338983 |
| AFTER |  | 8 | 0,0597014925 | 0,0447761194 | 1,70338983 | 1,74782609 |
| BEFORE |  | 3 | 0,0696202532 | 0,0646766169 | 0,887640449 | 0,971014493 |
| BEFORE |  | 3 | 0,0646766169 | 0,104712042 | 0,971014493 | 1,08522727 |
| TRAINING |  | 3 | 0,104712042 | 0,0646766169 | 1,08522727 | 1,0923913 |
| TRAINING |  | 13 | 0,0298507463 | 0,039800995 | 0,990147783 | 0,877729258 |
| AFTER |  | 13 | 0,039800995 | 0,0597014925 | 0,877729258 | 1,06349206 |
| AFTER |  | 13 | 0,0597014925 | 0,039800995 | 1,06349206 | 1,13559322 |
| TRAINING |  | 10 | 0,0298507463 | 0,0348258706 | 1,23312883 | 1,2484472 |
| AFTER |  | 10 | 0,0348258706 | 0,0209424084 | 1,2484472 | 1,17177914 |
| AFTER |  | 10 | 0,0209424084 | 0,0149253731 | 1,17177914 | 1,36734694 |
| BEFORE |  | 15 | 0,208955224 | 0,109452736 | 0,44966443 | 0,465277778 |
| BEFORE |  | 15 | 0,109452736 | 0,0945273632 | 0,465277778 | 0,492647059 |
| TRAINING |  | 15 | 0,0945273632 | 0,164179104 | 0,492647059 | 0,451685393 |
| TRAINING |  | 18 | 0,129353234 | 0,0597014925 | 0,663366337 | 0,598214286 |
| AFTER |  | 18 | 0,0597014925 | 0,105820106 | 0,598214286 | 0,726923077 |
| AFTER |  | 18 | 0,105820106 | 0,134328358 | 0,726923077 | 0,747211896 |
| TRAINING |  | 4 | 0,114427861 | 0,0547263682 | 0,674496644 | 0,779069767 |
| AFTER |  | 4 | 0,0547263682 | 0,0348258706 | 0,779069767 | 0,725631769 |
| AFTER |  | 4 | 0,0348258706 | 0,0447761194 | 0,725631769 | 0,782101167 |
| BEFORE |  | 6 | 0,00995024876 | 0,0149253731 | 1,09836066 | 1,23312883 |
| BEFORE |  | 6 | 0,0149253731 | 0,0149253731 | 1,23312883 | 1,09836066 |
| TRAINING |  | 6 | 0,0149253731 | 0,00497512438 | 1,09836066 | 1,11666667 |
| TRAINING |  | 16 | 0,104477612 | 0,0621761658 | 0,8375 | 0,946078431 |
| AFTER |  | 16 | 0,0621761658 | 0,0945273632 | 0,946078431 | 1,03076923 |
| AFTER |  | 16 | 0,0945273632 | 0,0746268657 | 1,03076923 | 0,934883721 |
| BEFORE |  | 11 | 0,109452736 | 0,07960199 | 0,830578512 | 0,851694915 |
| TRAINING |  | 11 | 0,07960199 | 0,0696517413 | 0,851694915 | 1,03608247 |
| AFTER |  | 11 | 0,0696517413 | 0,149253731 | 1,03608247 | 0,934883721 |
| BEFORE |  | 9 | 0,109452736 | 0,184079602 | 0,383587786 | 0,538873995 |
| BEFORE |  | 9 | 0,184079602 | 0,199004975 | 0,538873995 | 0,603603604 |
| TRAINING |  | 9 | 0,199004975 | 0,134328358 | 0,603603604 | 0,575931232 |
| BEFORE |  | 12 | 0,164179104 | 0,218905473 | 0,281512605 | 0,327895595 |
| BEFORE |  | 12 | 0,218905473 | 0,179104478 | 0,327895595 | 0,331683168 |
| TRAINING |  | 12 | 0,179104478 | 0,1875 | 0,331683168 | 0,312195122 |
| TRAINING |  | 1 | 0,174129353 | 0,165745856 | 0,386538462 | 0,397802198 |
| AFTER |  | 1 | 0,165745856 | 0,213930348 | 0,397802198 | 0,472941176 |
| AFTER |  | 1 | 0,213930348 | 0,109452736 | 0,472941176 | 0,505025126 |
| BEFORE |  | 17 | 0,0298507463 | 0,0348258706 | 1,26415094 | 1,21084337 |
| TRAINING |  | 17 | 0,0348258706 | 0,0447761194 | 1,21084337 | 1,29677419 |
| AFTER |  | 17 | 0,0447761194 | 0,07960199 | 1,29677419 | 1,34 |
| BEFORE |  | 14 | 0,119402985 | 0,169154229 | 0,552197802 | 0,674496644 |
| TRAINING |  | 14 | 0,169154229 | 0,158823529 | 0,674496644 | 0,833333333 |
| AFTER |  | 14 | 0,158823529 | 0,195266272 | 0,833333333 | 0,804761905 |
| BEFORE |  | 20 | 0,167539267 | 0,188235294 | 1,01595745 | 1,08280255 |
| BEFORE |  | 20 | 0,188235294 | 0,134328358 | 1,08280255 | 1,05789474 |
| TRAINING |  | 20 | 0,134328358 | 0,134328358 | 1,05789474 | 0,995049505 |
| BEFORE |  | 5 | 0,0842105263 | 0,07960199 | 0,811965812 | 0,889380531 |
| TRAINING |  | 5 | 0,07960199 | 0,0497512438 | 0,889380531 | 0,897321429 |
| AFTER |  | 5 | 0,0497512438 | 0,0837696335 | 0,897321429 | 0,93627451 |
|  |  |  |  |  |  |  |
